# Case report: Benign and malignant tumors in adult patients with neurofibromatosis type 1: a comprehensive case series from a large oncologic reference center

**DOI:** 10.3389/fonc.2023.1291286

**Published:** 2024-01-08

**Authors:** Silvia Vidal-Millan, Zyanya Lucia Zatarain-Barrón, Kena Daza-Galicia, Daniela Shveid Gerson, Pavel Salvador Pichardo-Rojas, Alejandro Salazar-Pigeon, Talia Wegman-Ostrosky

**Affiliations:** ^1^ Hereditary Cancer Clinic, Instituto Nacional de Cancerologia, Mexico City, Mexico; ^2^ Department of Math and Science, Arkansas State University Querétaro, Querétaro, Mexico; ^3^ Subdirection of Basic Research, Instituto Nacional de Cancerología, Mexico City, Mexico; ^4^ Faculty of Higher Studies Iztacala, Universidad Nacional Autónoma de México, Mexico City, Mexico; ^5^ Oncology Department, ABC Medical Center, Mexico City, Mexico; ^6^ The Vivian L. Smith Department of Neurosurgery, The University of Texas Health Science Center at Houston McGovern Medical School, Houston, TX, United States; ^7^ Plan of Combined Studies in Medicine (PECEM-MD/PhD), Faculty of Medicine, Universidad Nacional Autónoma de México, Mexico City, Mexico

**Keywords:** neurofibromatosis type 1, adults, breast cancer, malignancies, malignant peripheral nerve sheath tumors, Hispanics

## Abstract

**Purpose:**

Neurofibromatosis type 1 (NF1) is a complex, multisystem disorder that is characterized, among other features, by a higher risk of developing benign and malignant tumors. Despite NF1 being one of the most common autosomal dominant genetic disorders, data from adult individuals in several world regions remain elusive, including Hispanics.

**Methods:**

The present is a retrospective cohort study conducted among adult patients with a confirmed diagnosis of NF1 who attended a single cancer-reference center, the Instituto Nacional de Cancerología in Mexico City from 2001 to 2021. Data were extracted from electronic health records and collected in an anonymous database by an NF1-expert physician in order to obtain demographic characteristics and detailed information regarding the development of tumors among this patient subgroup. All patients with malignant tumors or with benign tumors, which severely affected their quality of life, were included in this study.

**Results:**

Patient records were reviewed from 2001 to 2021. A total of *N* = 29 patients met the criteria, with a higher proportion of female compared with male subjects [*N* = 22 (75.9%) vs. *N* = 7 (24.1%)]. Patients had a mean age at diagnosis of tumors of 32.2 years (*SD* = 11.2 years). In terms of malignant neoplasms, the most frequent malignant tumor presented by patients in this cohort was malignant peripheral nerve sheath tumors (*N* = 7, 24.1%), this was followed by breast cancer (*n* = 4, 13.8% among all patients, 18.2% among female patients). Other tumors also identified in this cohort included melanoma, gastrointestinal stromal tumors, and rectal cancer.

**Conclusion:**

In Mexico, patients diagnosed with NF1 develop diverse tumors as adults. As described in other studies, the most frequent malignant tumor in this patient population is the malignant peripheral nerve sheath tumor. Further studies are required to increase the scarce information available for adult Hispanics with NF1.

## Introduction

**Figure 1:**
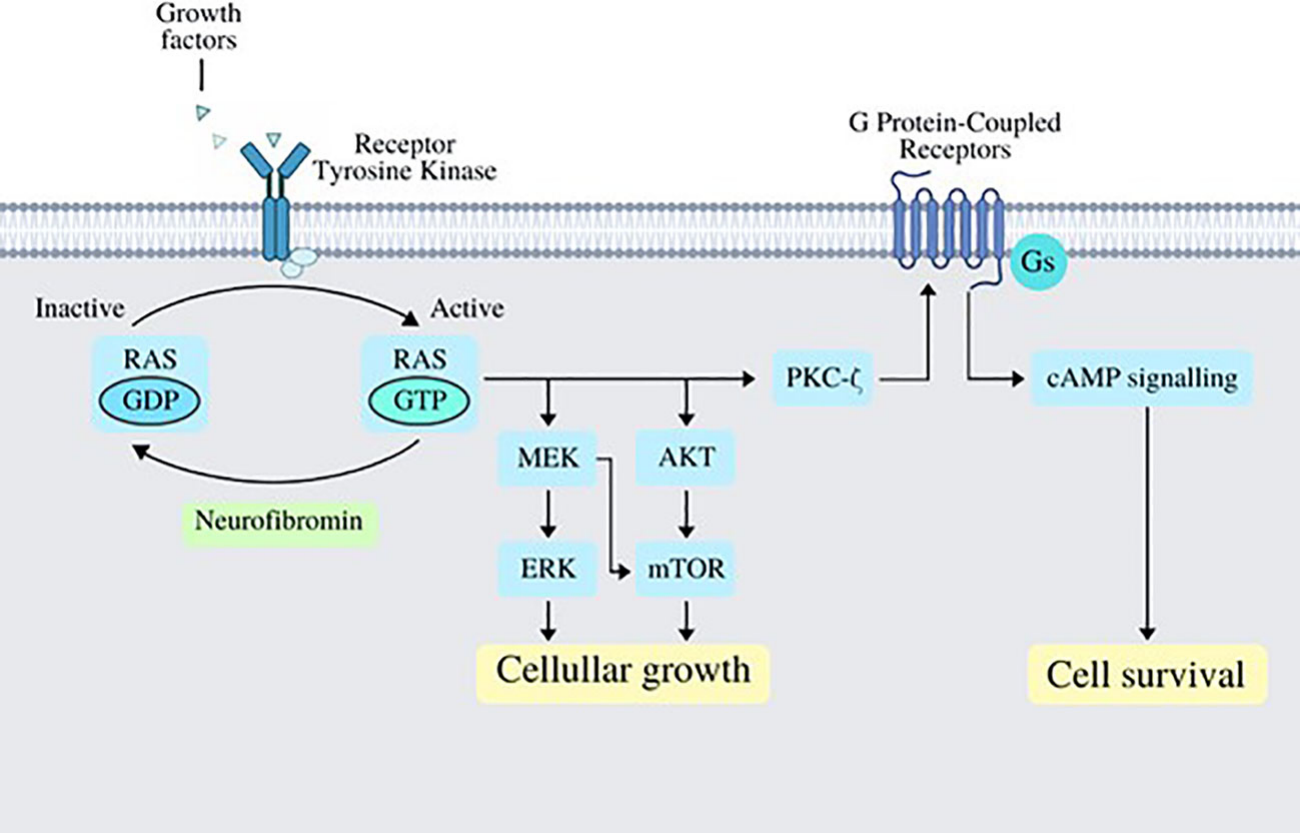
Several growth factors work by binding its specific tyrosine kinase receptors, which in turn activate RAS via phosphorylation of GDP-bound RAS turning it into GTP-bound RAS; this step is normally antagonized and regulated by neurofibromin. Once RAS has acquired its active form, it works by activating MEK/ERK and AKT/mTOR signaling pathways, which end up in cellular growth; RAS also activates PKC-ζ, which works as a GPCR activator, which in turn activates cAMP signaling and promoting cell survival. cAMP, cyclic AMP, adenosine monophosphate; GPCR, G protein–coupled receptors; ERK, extracellular signal-regulated kinase; mTOR, mechanistic target of rapamycin.

Neurofibromatosis type 1 (NF1) is a complex disorder that affects multiple systems. The disease arises from the loss of function in the gene that codes for NF1, an important tumor suppressor. Patients diagnosed with NF1 present a predisposition for tumor development, and although the disease is inherited in an autosomal dominant pattern, up to one-half of patients with NF1 have *de-novo* variants, highlighting the mutation rate present in the *NF1* gene, which is among the highest known for any human gene (1). Individuals with NF1 are prone to develop diverse benign and malignant tumors, including neoplasms of the central and peripheral nervous system. This predisposition is highly relevant in the clinical setting, in fact, neoplastic diseases represent the most common cause of death among patients with NF1 (1, 2). In the context of cancer, the loss of normal function of neurofibromin via genetic mutation results in heightened cell proliferation and migration, predisposing NF1 patients to malignant growths (3). Neurofibromin can be found in many cell types across the body; it works as a negative regulator of cell growth and survival. Growth factors normally bind tyrosine kinase receptors which, in turn, activate the RAS signaling pathway by accelerating the conversion of inactive Guanosine diphosphate (GDP)-bound RAS to its active Guanosine triphosphate (GTP)-bound form; this step is normally antagonized by neurofibromin’s function (4, 5). Activating the RAS signaling system will consequently promote AKT and/or MEK activity, which will subsequently produce biologic responses that promote cellular growth. Furthermore, RAS can enhance the production of cyclic adenosine monophosphate (cAMP) via protein kinase C-ζ (PKCζ) following the activation of G protein–coupled receptors (GPCRs), which may promote cellular survival (**Figure 1**).

The main type of cancers arising in patients with this disorder includes optic pathway gliomas, non-optic glioma brain tumors, malignant peripheral nerve-sheath tumors (MPNSTs), gastrointestinal stromal tumors (GISTs), breast cancer, leukemia, pheochromocytoma, duodenal carcinoid tumor, and rhabdomyosarcoma (1, 2).

The predisposition to develop specific neoplasms among patients with NF1 is widely recognized, although the specific role played by germline mutations and the predictive role they might have as to which patients are more likely to develop malignant neoplasms has long been under assessment (6). Furthermore, current evidence driving recommendations and follow-up guidelines for patients with NF1 stem largely from Caucasian populations or patients living in developed regions. Interestingly, one study included in a previous meta-analysis, which sought to assess the prevalence of NF1 across the available literature provided evidence from Hispanics in Cuba, highlighting an estimated prevalence of 1:1141 compared with the pooled NF1 prevalence of 1:3164. In this case, the results might represent a higher identification from a screening type of study, although the authors do conclude a high prevalence in the group studied in Cuba (7). Additionally, several studies have since emerged that highlight ethnic and racial differences in terms of frequency of diverse neoplasms in patients with NF1, including malignant peripheral nerve sheath tumors and brain tumors in pediatric patients (8, 9). The data support differences in the frequencies for these types of tumors depending on race and ethnicity, nonetheless, the studies have been performed in U.S.-based registries and, therefore, do not reflect data from Hispanics living outside of the United States, including Latin America.

In Mexico, and other Latin American countries, research aiming to understand the characteristics and challenges faced by patients with NF1 has lagged behind. Currently, such areas lack studies to assess the regional prevalence and incidence of this disease, potentially hampering this healthcare setting. Furthermore, the differences in the frequency and characteristics of malignancies among adults with NF1 have not been previously assessed in the region.

The present study sought to describe the types of neoplasms developed by patients diagnosed with NF1 and who received treatment and follow-up at a large, cancer-reference center in Mexico City.

## Materials and methods

### Patients and data collection

All patients who had a confirmatory diagnosis of NF1 and who attended the Instituto Nacional de Cancerología (INCan) in Mexico City from 2001 to 2021 were eligible to be included in this study. Patient records were reviewed to identify subjects who met criteria and who were admitted for treatment at INCan from a diagnosis of a malignant tumor or a benign tumor that severely compromised quality of life, defined as requiring palliative care for pain control or specialized surgical treatment. Demographic and clinical data were collected: age, gender, family history of malignant tumors, family history of NF1, age at malignant onset, tumor location, histopathological characteristics, and treatment. Written informed consent was obtained for use of images and radiological studies.

Anonymized data were collected in a database by a geneticist with expertise in NF1 associated malignancies. For descriptive purposes, continuous variables were summarized as arithmetic means, and standard deviations (SDs). Categorical variables were reported as proportions. Overall survival was calculated using the Kaplan–Meier method and defined as the time between histopathological diagnosis of the benign or malignant tumor considered for inclusion in this study, until death by any cause or loss to follow up. All the analyses were performed using SPSS version 23.0 (SPSS, Inc., Chicago, IL, USA).

## Results

Twenty-nine patients met the inclusion criteria, 22 (75.9%) were female and seven were male (24.1%) (**Tables 1**, **2**). The age of diagnosis of NF1 syndrome ranged from 0 to 29 years; all the patients included in this cohort were adults at the time they were evaluated at the INCan, although some were included because of recurrent tumors and so the age at which the primary tumor was initially diagnosed was recorded. The mean age at the time of diagnosis of benign or malignant tumor was 32.2 years (*SD* = 11.2, range from 16 to 56 years). Interestingly, the mean age at diagnosis was lower in patients who presented with benign tumors (*N* = 14, 28.1 years, *SD* = 9.2) compared with those who presented with malignant tumors (N = 15, 35.9 years, *SD* = 11.9).

**Table 1:** Case descriptions.

Patient number	Sex	Last known status	Tumor type	Tumor site	Age at diagnosis	Surgical treatment	Systemic treatment	Palliative treatment	Additional relevant clinical data
NON-MALIGNANT TUMORS									
2	Female	Alive	PNST	Face	16	Yes	NR	NR	Patient with a recurrent neurofibroma of the left face, which was initially diagnosed at the age of 16, due to the extension the patient had to undergo an enucleation of the left eye.
3	Female	Alive	PNST	Gluteal	50	NR	NR	NR	Patient with a large gluteal PNST which severely affected quality of life.
4	Female	Alive	PNST	Lumbosacral region, popliteal region, vulvar area and costal region.	30	Yes	No	Yes	Patient underwent surgical resections for several lesions; however, she developed severe pain which was difficult to manage. She thereafter refused surgical treatment for the lesions located in the vulva due to pain and lack of clinical benefit as assessed by the patient from previous interventions. She was referred to the PCU for pain management.
6	Female	Deceased	PNST	Para-pharyngeal region	25	Yes	No	Yes	Patient presents with a recurrent tumor which she has had previously undergone surgical treatment for in three occasions. She undergoes an additional surgical resection and receives radiotherapy (60 Gy). The patient is referred to PCU for palliative services and pain management and died three years following the last surgical intervention.
7	Male	Alive	PNST	Mediastinum and occipital region	16	Yes	No	NR	Patient undergoes surgery via a posterolateral thoracotomy to remove mediastinal lesion; additionally undergoes neurosurgery for occipital lesion.
8	Female	Alive	Multiple PNST	Lumbosacral region	30	No	No	NR	Patient with multiple lumbosacral schwannomas, in addition to lesions on her wrist and facial area. She undergoes surgical resection but solely for the lesions on her wrist, other lesions are not subject to surgical resection.
9	Male	Alive	PNST	Cervical region	29	Yes	No	NR	Patient with a recurrent tumor with a previous resection at the age of 23 and a follow-up resection at INCan one year after. Patient refers his grandfather on his father´s side presented with pancreatic cancer.
10	Female	Alive	PNST	Right portion of the neck	16	No	No	NR	Patient presents a large neurofibroma on her rick neck which is considered non-resectable. She undergoes treatment with radiotherapy (30 Gy) without tumor response. A follow-up magnetic resonance showed multiple lesions extending from the base of the tongue towards the cervical region. No further information is available.
11	Female	Alive	PNST	Iliac fossae	22	Yes	No	NR	Patient is referred to INCan for surgical management of recurrent neurofibromas on her right forearm and iliac fossae which had been previously resected at the age of 2 and then again at the age of 16. She presents for further surgical management.
12	Female	Alive	PNST	Abdomen	32	Yes	No	NR	Recurrent PNST. Patient has had multiple previous surgical resections since the age of six, she is referred to INCan due to imaging studies showing a PNST in the abdominal wall, which underwent surgical resection.
18	Female	Alive	Pleomorphic adenoma	Left portion of the neck	38	Yes	No	No	Surgical resection in 2010, patient referred three previous resections in a period of 20 years.
26	Male	Alive	PNST	Forearm	29	No	No	Yes	Large (>15 cm) tumor caused muscular atrophy, and largely extended into the flexor compartment. Patient developed depressive disorder. Required a stellate ganglion block and infiltration of the trapezium muscle.
27	Female	Alive	PNST	Gluteal	29	No	No	No	Large neurofibroma in gluteal region (>15 cm). Patient was scheduled for treatment but after the initial biopsy was performed she was referred to an obstetrics facility as she reported since then she had become pregnant.
28	Female	Alive	PNST	Right parotid	32	Yes	No	No	Neurofibroma in the right parotid region. Neoplasm of diffuse neural origin which extends over skin annexes and adipose tissue in the right parotid region. Previous resection of fibromas located on the facial region and right wrist in 2020.
MALIGNANT TUMORS									
1	Female	LFU	Acute myeloid leukemia M2	Hematological	34	No	Yes	NR	Patient had previously developed a peripheral nerve sheath tumor on the left side of her neck. She received 2 cycles of cytarabine and daunorubicin; malignancy was treatment refractory.
5	Female	Deceased	Metastatic MPNST	Left thigh	25	No	No	Yes	Patient presented with a MPNST on her left thigh at 25 years of age, in addition to a neurofibroma of the sciatic nerve. She underwent surgical resection for both tumors, however one year later she presented with lung metastases and refused surgery due to previous complications. She died at the age of 26.
13	Female	Deceased	MPNST	Retroperitoneum	23	Yes	NR	NR	Patient is the twin sister of patient number 5. She was treated via surgical resection, but the tumor progressed subsequently, the patient died four months after tumor progression.
14	Male	Alive	MPNST	Retroperitoneum	37	No	Yes	NR	Patient refers a brother and sister also diagnosed with NF1. He was diagnosed 8 years before referral to INCan due to a retroperitoneal MPNST which extended from the renal hilum to the pelvic floor. He received systemic treatment based on cisplatin/Adriamycin and ifosfamide/Adriamycin.
15	Female	LFU	MPNST	Gluteal	29	NR	NR	NR	Patient presents with a large gluteal MPNST which compromises quality of life.
16	Female	LFU	MPNST	Sacroiliac region	19	Yes	No	Yes	Patient with multiple tumors throughout her lifetime, refers 10 previous surgical procedures, including enucleation of her left eye at the age of one. She is referred to INCan for a sacroiliac MPNST with lung metastases. She was referred to palliative care for pain management.
17	Male	Deceased	MPNST (Malignant triton)	Occipital-cervical region	32	Yes	No	Yes	Patient presents with a malignant high grade neurogenic sarcoma. At the time of referral to INCan he had already undergone 5 previous surgical resections at a different healthcare facility, but at age 32 he was diagnosed with tumor progression, including mediastinal metastasis. The patient was assessed for palliative care but died one month after his last consult.
19	Female	Deceased	Bilateral metachronous infiltrating canalicular carcinoma of the breast.	Breast	49	Yes	Yes	No	Bilateral mastectomy was performed (2001 and 2008). Patient received neoadjuvant chemotherapy and chemoradiotherapy thereafter.
20	Female	Alive	Infiltrating canalicular carcinoma of the breast; Endometrial cancer	Breast; uterus	46	Yes	NR	No	Patient has a first biopsy report from 2006 which shows microscopic ductal hyperplasia with atypia. In 2011 she is reported to have an infiltrating canalicular carcinoma. Patient underwent a simple right mastectomy in 2012. Following, the patient is diagnosed with endometrial cancer in 2015 and underwent a hysterectomy and salpingo-oophorectomy. She and patient 19 are sisters, both tested for *BRC1/2* gene mutations, which were negative.
21	Female	LFU	Conjunctival melanoma	Left eye	30	Yes	No	Yes	Recurrent tumor, initial surgery at age 11, recurrence at age 31. Treated with exenteration of the left eye and conjunctiva, tumor dimensions were 1.5 x 2.0 cm. Pain required management by the PCU.
22	Female	Alive	Gastrointestinal stromal tumor	Small intestine	56	Yes	Yes	No	Patient presented with sudden pelvic pain at age 56, underwent an exploratory laparotomy which identified a sigmoid perforation, an iliac abscess and two tumors in the jejunum. IHC profile was C-KIT (+), CD34 (+), vimentin (+) and BCL2 (+). Treatment was initiated with imatinib. Patient with stable disease 31 months after initiating therapy.
23	Male	LFU	Adenocarcinoma	Colorectal region	52	Yes	Yes	No	Patient presented with neoplasm at age 52; received neoadjuvant chemotherapy with capecitabine and radiotherapy (50.6 Gy) before being scheduled for a surgical resection. The patient was LFU shortly after.
24	Female	LFU	Stage IVB breast cancer (lung metastases)	Breast	31	No	No	Yes	NF1 diagnosis made following breast cancer presentation at young age and referral to medical genetics service. At 18 months following the initial diagnosis patient was referred to PCU with a poor prognosis.
25	Female	LFU	Infiltrating lobular carcinoma of the breast	Breast	51	NR	NR	NR	Female patient diagnosed with a breast carcinoma who was LFU shortly after diagnosis.
29	Male	Alive	MPNST	Paravertebral	25	Yes	No	Yes	Sister and mother deceased from unspecified cancer; patient was left with paresthesia of the left leg after surgical resection.

PCU, Palliative Care Unit; LFU, Loss to follow up; NR, Not reported; PNST, Peripheral nerve sheath tumor; MPNST, Malignant peripheral nerve sheath tumor; INCan, National Cancer Institute of Mexico.

**Table 2:** Characteristics and differences between patients with benign and malignant tumors.

Characteristic	Total (N=29)	Benign (N=14)	Malignant (N=15)
Sex			
Male	7 (24.1%)	3 (21.4%)	4 (26.7%)
Female	22 (75.9%)	11 (78.6%)	11 (73.3%)
Tumor site			
Head and neck	9 (31%)	7 (50.0%)	2 (13.3%)
Abdomen	3 (10.3%)	1 (7.1%)	2 (13.3%)
Thorax	6 (20.7%)	1 (7.1%)	5 (33.3%)
Lumbosacral region	3 (10.3%)	2 (14.3%)	1 (6.7%)
Glute	3 (10.3%)	2 (14.3%)	1 (6.7%)
Extremities	2 (6.9%)	1 (7.1%)	1 (6.7%)
Tumor type			
PNST	13 (44.8%)	13 (92.9%)	NA
Pleomorphic adenoma	1 (3.4%)	1 (7.1%)	NA
MPNST	7 (24.1%)	NA	7 (46.7%)
Breast cancer	4 (13.8%)	NA	4 (26.7%)
Colorectal cancer	1 (3.4%)	NA	1 (6.7%)
Melanoma	1 (3.4%)	NA	1 (6.7%)
Other	2 (6.9%)	NA	2 (13.3%)
Recurrent tumors	6 (20.7%)	5 (35.7%)	1 (6.7%)
Last known patient status			
Alive	17 (58.6%)	13 (92.9%)	4 (26.7%)
Deceased	5 (17.2%)	1 (7.1%)	4 (26.7%)
Loss to follow up	7 (24.1%)	0 (0.0%)	7 (46.7%)
Treatment type*			
Systemic therapy	5 (17.2%)	0 (0.0%)	5 (33.3%)
Surgical resection	18 (62.1%)	9 (64.3%)	9 (60.0%)
Palliative care	9 (31.0%)	3 (21.4%)	6 (40.0%)
Family history of cancer	11 (37.9%)	4 (28.6%)	7 (46.7%)
Mean age at the time of diagnosis (SD)	32.2 (11.2)	28.1 (9.2)	35.9 (11.9)

NA, Not applicable; PNST, Peripheral nerve sheath tumor; MPNST, Malignant peripheral nerve sheath tumor; SD, Standard deviation. *Some patients received more than one type of intervention.

The most common malignancy diagnosed in this cohort was malignant peripheral nerve sheath tumors (*N* = 7, 24.1%) (**Table 1**), followed by breast cancer (N = 4, 13.8%). Other neoplasms, namely, GISTs (*N* = 1), melanoma (*N* = 1), and leukemia (*N* = 1) were also identified in this patient series.

When grouping the cases by tumor site, the most frequent tumor location was head and neck (*N* = 9, 31%), followed by the thoracic area (*N* = 6, 20.7%). Other regions affected included the abdomen (*N* = 3, 10.3%), lumbosacral region (*N* = 3, 10.3%), and gluteal area (*N* = 3, 10.3%). It is important to highlight that, among the 14 patients with benign tumors included in this series, 50% presented with tumors in the head and neck region (*N* = 7), which underscores the considerable burden placed on patients by benign tumors located in this area.

In terms of treatment, patients included in this series received systemic therapy (*N* = 5, all of whom were categorized as malignant neoplasms), an additional 62.1% received surgical therapy [distributed similarly between the benign (*N* = 9] and the malignant (*N* = 9) subgroups], and an additional 31% required specialized management for pain by the palliative care unit, most of these patients with a malignant neoplasm diagnosis (*N* = 5).

**Figure 2:**
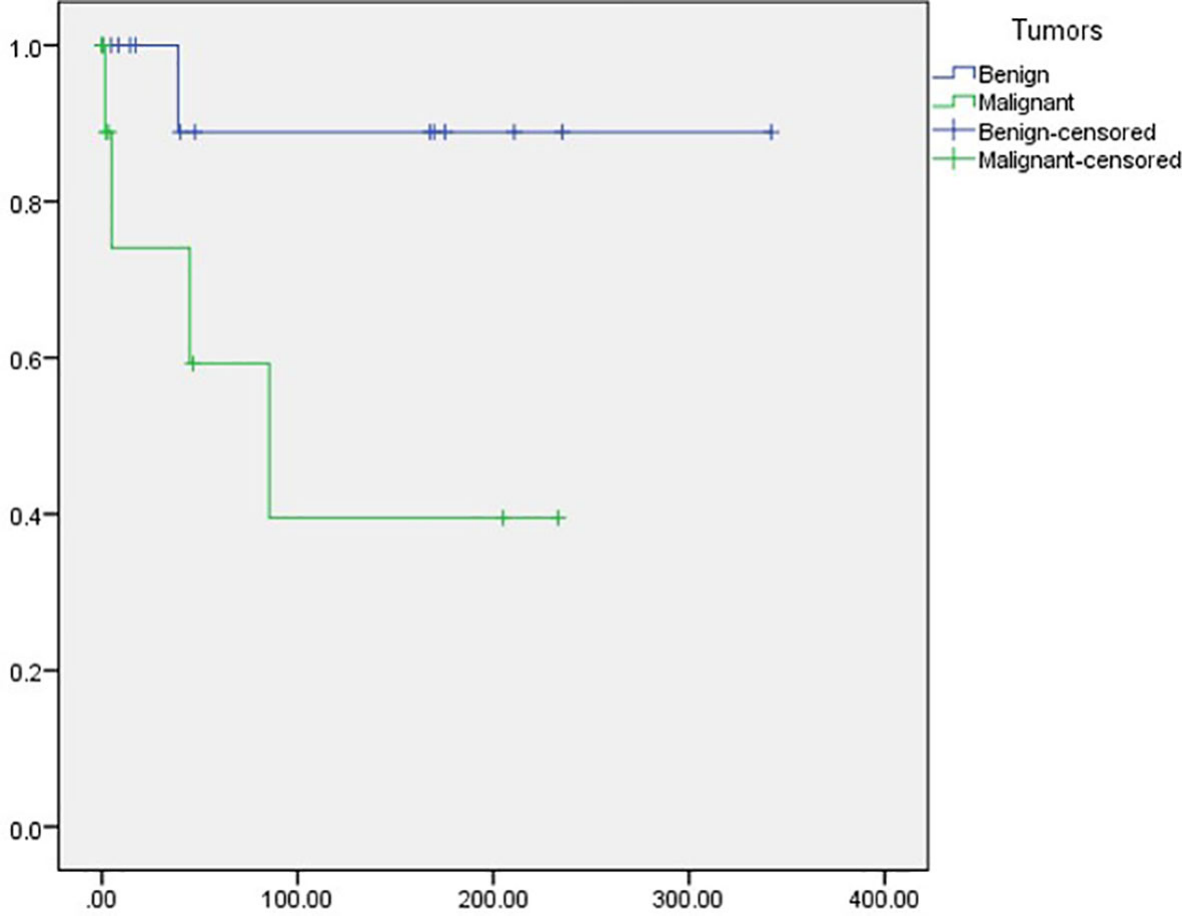
Overall survival among patients with benign and malignant tumors.

In terms of outcomes, we recorded the last known patient status available from the records. Among the included patients, 58.6% (*N* = 17) were reported as alive, most being patients with benign tumors (*N* = 13, 92.9%) compared with those diagnosed with malignant neoplasms (*N* = 4, 26.7%). A total of five patients had died (17.2%), including mostly patients with malignant (*N* = 4; 26.7%) versus benign (*N* = 17.1%) tumors. Last, seven patients had been lost to follow up, all included in the malignant tumor subgroup. We identified a mean Overall survival (OS) of 246.6 months (95% CI 175.5–317.6). Importantly, patients diagnosed with malignant tumors had a significantly shorter OS compared with those diagnosed with benign tumors [116.5 months (95%CI 40.4–192.6) vs. 308.4 (95%CI 246.1–370.6); *p* = 0.046] (**Figure 2**).

### Brief description of selected cases

Patient 5 and patient 13 are twin sisters who both present with NF1. Patient 5 had an MPNST on her left thigh at 25 years of age, in addition to a neurofibroma of the sciatic nerve. Both tumors underwent surgical resection; nonetheless, 1 year following the initial diagnosis, lung metastases were identified. The patient died at 26 years of age. Meanwhile, patient 13 is a female who developed an MPNST in the retroperitoneum. She received treatment via surgical resection; however, the tumor progressed and the patient subsequently died 4 months after tumor progression.

Patient 17 was a male patient diagnosed at 29 years of age with a malignant triton of the occipito—cervical region. He received treatment with multiple surgical resections; at age 32 years, the tumor progressed, with dimensions of 15 cm x 20 cm. Furthermore, imaging studies showed a mediastinal metastasis. The patient died a month later.

Patients 19 and 20 are two sisters with NF1 who presented with breast cancer. Patient 20 was diagnosed at 46 years of age with an infiltrating canalicular carcinoma; the tumor was positive for hormone receptors and negative for the Her2neu receptor. Patient 19 was diagnosed at 49 years of age with bilateral metachronous infiltrating canalicular carcinoma with an *in-situ* ductal carcinoma component, SRB 9, and triple negative phenotype. Both patients were tested for the mutations in the *BRC1/2* gene; complete sequencing and complex rearrangements were negative.

## Discussion

In this study, we present the first case series of adult patients with NF1 in Mexico treated at a large national reference center for diverse types of tumors; in the 20-year-period established in this protocol, we identified 29 patients with NF1 that were treated at INCan. In this cohort, 48% presented benign tumors that compromised their quality of life and required specialized treatment, and 52% presented malignant tumors. The predominant malignancy identified in this group was malignant peripheral nerve sheath tumors, with breast cancer being the second most frequently diagnosed. Additionally, various other neoplasms were observed in this patient series, such as colon cancer, GISTs, melanoma, and leukemia. The mean age for benign tumors was 28.1 years and for malignant tumors was 35.9. These data support the concept of NF1 as a cancer predisposition syndrome that not only affects children but also adults. Results have shown that patients with NF1 develop neoplasms at a younger mean age and more frequently compared with the general population (10, 3, 6).

Currently, there is no information regarding the incidence or prevalence of NF1 in Mexico, and data from Latin America are also very limited. In a recent systematic review and meta-analysis (11), the authors included a study from Cuba that sought to present data from the region; in this study, the authors identified a prevalence rate of NF1 of 1:1141 (7). It is important to note that this information came from a study among pediatric patients and using a screening strategy.

In our present case series, the 29 adult patients with NF1 and diverse tumor types identified over a 20-year period represents an enormous sub-estimation of the population facing this condition and who potentially are left without specialized care. If considering the population of Mexico (126.7 million) and the currently reported worldwide prevalence (1:3000), one might estimate that there are approximately 42,233 people with NF1 living in Mexico. Among these individuals, approximately 35.5% are eligible for treatment at National Health Institutes, including INCan, meaning that 14,992 NF1 patients are available for nationwide reference to this center once they reach adulthood. Considering a lifetime risk of developing MPNST for NF1 patients of 10% and narrowing to a 20-year study period, an estimated 375 MPNST cases would be expected. This contrasts severely with the seven cases reported in this series, which could potentially mean that only 1.9% of the patients with NF1 who develop MPNST and are eligible for treatment at INCan are being detected, referred, treated, and followed, potentially leaving a staggering 98% of these patients undetected.

As can be inferred by these numbers, many patients face the challenge of Mexico´s scarce medical genetic services, which includes one specialist for 525,000 inhabitants. This is far from the current U.S./U.K. standard of 1:100,000 inhabitants. Furthermore, the distribution of specialists and services is highly unequal, most being located in the capital, Mexico City, leaving large rural areas unattended and many patients without an adequate referral system. All of these considerations, together with the potential loss to follow up for patients with NF1 who are discharged from pediatric care facilities after turning of age, contribute to the immense gap in the expected number of cases versus the actual cases identified in this study and highlight the urgent need for improvements in the adequate detection, referral, treatment, and follow-up of adult patients with NF1 in Latin America.

Compared with the general population, patients with NF1 have a considerable decrease in life expectancy (Rasmussen, 2001). In a study that retrieved information from 1895 NF1 patients between 1980 and 2006, the authors observed a significantly increased mortality as assessed by the Standardized Mortality Ratio, compared to the general population. The excess mortality occurred among patients between the ages of 10–20 years and 20–40 years and mainly stemmed from MPNST (12).

The leading causes of NF1-associated deaths are related with malignant neoplasms of connective and other soft tissues and brain (1). It is well known that NF1 is a risk factor for tumors of the central and peripheral nervous systems, but other types of neoplasms have been shown to have significantly elevated risks among patients diagnosed with NF1 compared with the general population, namely, esophageal cancer (RR: 3.3) stomach cancer (2.8), colon cancer (2.0), liver cancer (3.8), biliary tract cancer (8.2), pancreatic cancer (3.4), lung and bronchus neoplasms (3.0), malignant melanoma (3.6), non-melanoma skin cancer (1.6), thyroid gland cancer (4.9), breast cancer (2.3), and ovarian cancer (3.7) among others (3). In a large cohort study published in 2021, the prevalence of neoplasia among 1,607 patients with NF1 was evaluated. The NF1 patient group was compared with estimates obtained from the Surveillance, Epidemiology, and End Results database, and results highlight that patients with NF1 develop low-grade gliomas, high-grade gliomas, MPNST, and breast cancer at a younger age compared with the general population.

Since several decades ago, data have shown the effect of malignant neoplasms on survival of patients diagnosed with NF1. In a study performed by Sorensen et al., a cohort of 212 affected patients and families were identified and followed. Importantly, this study identified a decrease in survival rates in relatives with NF1 compared with general population. The study also presented data regarding the presence of malignant neoplasms or benign central nervous system tumors, and results demonstrated that patients with NF1 have a significantly higher risk of developing such tumors [Relative Risk 4.0 (95% CI 2.8–5.6)]. Furthermore, the study also showed that female relatives of NF1 patients had a higher risk of presenting neoplasms; this association was not ascertained for male relatives, highlighting a potential sex-dependent mechanism (13). Interestingly, 37.9% of the patients included in our study referred having had a close relative (i.e., mother, sister, father) die from diverse neoplasms; this proportion reached 46.7% among patients with malignant tumors included in this study. Additionally, our series present the case of two twin sisters who developed MPNST as well as two sisters with breast carcinomas and a patient who developed bilateral breast tumors, highlighting that NF1 is a tumor-developing syndrome, and that adult patients are at an elevated risk for several malignant neoplasms compared with the general population (10).

Currently, new approaches stemming from a precision medicine viewpoint hold tremendous promise for patients with NF1. Particularly, the MEK-inhibitor drug class has been recently successful for NF1-related conditions, including plexiform neurofibromas and low-grade gliomas. Selumetinib has since become the first Food and Drug Administration–approved targeted agent for NF1, but only for pediatric patients. In 2021, the drug received approval by Comisión Federal para la Protección contra Riesgos Sanitarios (COFEPRIS), the drug-regulatory agency of Mexico, following evidence from several phase 2 trials showing improvement in response, tumor pain intensity, and health-related quality of life (14, 15).

There are several potential implications for the data presented in this case series. First, it is important to note that breast cancer represents a frequently identified neoplasm among patients with NF1, and 18.2% of the female patients in this series presented with this diagnosis. The marked increased risk of breast cancer is well documented among this patient population, with median age of diagnosis reported at 46.9 years of age and a cumulative risk for contralateral breast cancer of 26.5% in 20 years (16). In this case series, four female patients developed breast cancer; one of them had bilateral cancer, with a mean age of 44.25 years.

**Figure 3:**
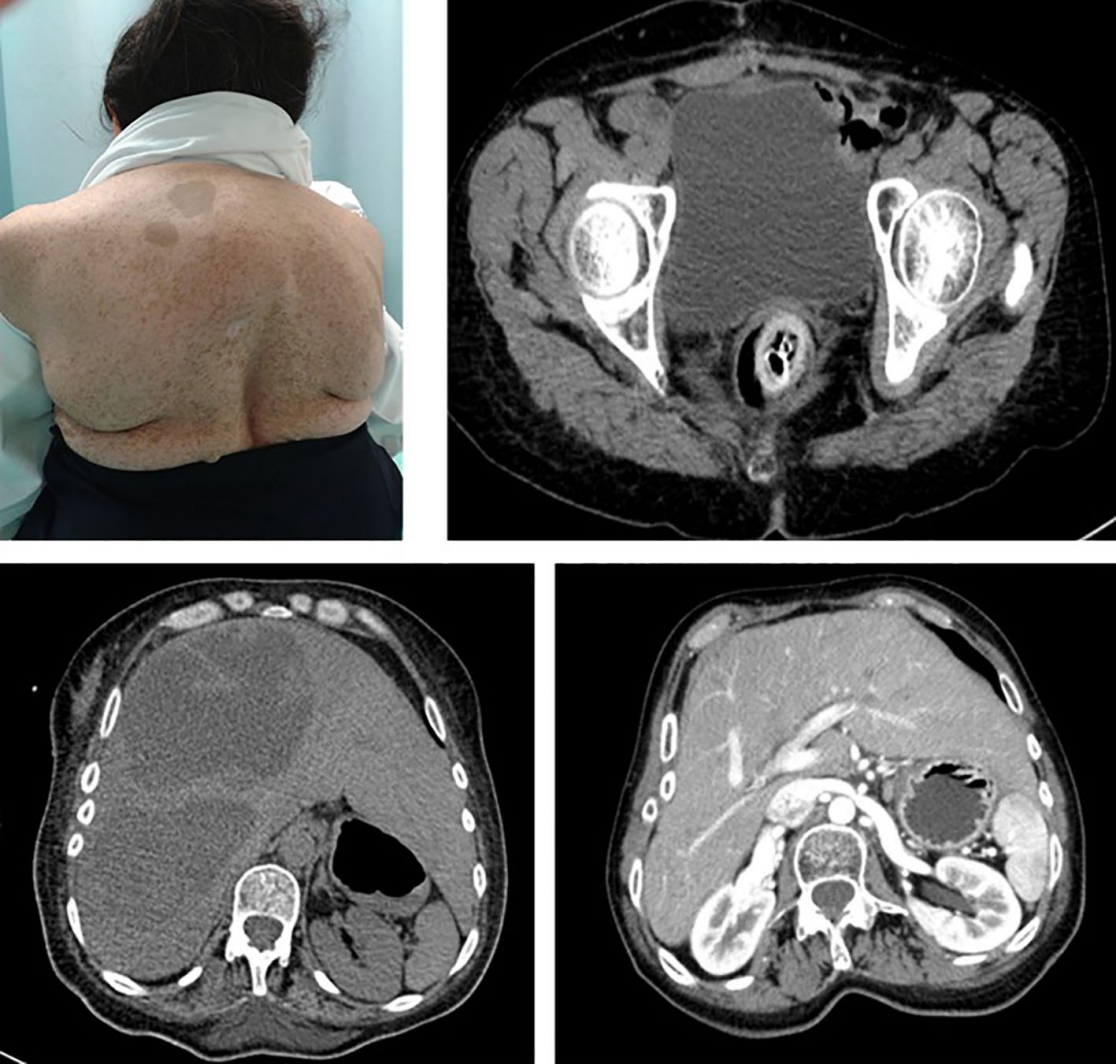
Patient 22 is a 56-year-old female subject with a NF1 diagnosis who presented with sudden pelvic pain. CT-imaging showed large liver lesions (arrows) which shifted the kidney to the pelvic fossae. An exploratory laparotomy was performed, and a sigmoidectomy and partial jejunum resection resulted. Histopathological report evidenced a multifocal GIST, with a C-KIT(+), CD34(+), vimentin (+), and BCL2(+) profile.

Among the other tumor subtypes identified in this cohort, GISTs have been previously studied as a common gastrointestinal manifestation of NF1, and a previous report from 70 NF1 patients in Sweden identified that up to 33% of patients develop GIST (17). These tumors differ from gastrointestinal neurofibromas and are typical of mid-adulthood among this patient population. Interestingly, GISTs arising in patients with NF1 differ in their molecular features from those occurring in patients without NF1 (18) (**Figure 3**).

Although several authors have described the malignant neoplasms present in NF1 patients in several regions (3, 6, 13, 19, 20), the current study represents the first to characterize the types of neoplasms diagnosed in adult patients with NF1 in Latin America, a largely unexplored patient subgroup. This information is greatly relevant in this context and could help to shed light on a very underserved population, which requires timely and up-to-date treatment options, as well as close follow-up. As of now, it may very well be that most NF1 patients requiring care in Mexico never reach a specialized healthcare provider.

Last, it is important to point out that the data from this study should be cautiously interpreted, especially considering the limitations inherently stemming from a study which has a retrospective design. Such limitation sinclude data based on recollecting information over a broad time period, from only one reference center which is available to a specific subset of the population in Mexico due to affiliation restrictions. Nonetheless, this represents the first information regarding adult patients with NF1 in Mexico and Latin America and adds to the available literature for patients within the region.

## Conclusions

Patients with NF in Mexico are susceptible to developing cancer not only during childhood but also as adults. Tumors derived from peripheral nerves are the most frequent but not the only types of malignant tumors emerging in this population. Importantly, the number of patients identified over a 20-year period is considerably low compared to estimations and previous studies, highlighting the need to improve the detection and referral for these patients so they can receive specialized care and follow-up. Healthcare facilities nationwide must improve awareness and implement sensible mechanisms for timely care of NF1 patients.

## Data availability statement

The raw data supporting the conclusions of this article will be made available by the authors, without undue reservation.

## Ethics statement

Ethical approval was not required for the study involving humans in accordance with the local legislation and institutional requirements. Written informed consent to participate in this study was not required from the participants or the participants’ legal guardians/next of kin in accordance with the national legislation and the institutional requirements. Written informed consent was obtained from the individual(s) for the publication of any potentially identifiable images or data included in this article.

## Author contributions

SV-M: Conceptualization, Data curation, Investigation, Methodology, Supervision, Writing – original draft, Writing – review & editing. ZZ: Investigation, Writing – original draft, Writing – review & editing. KD-G: Data curation, Formal analysis, Investigation, Methodology, Writing – original draft. DS: Conceptualization, Formal analysis, Investigation, Methodology, Writing – original draft. PP: Conceptualization, Data curation, Investigation, Methodology, Writing – original draft, Writing – review & editing. AS-P: Data curation, Investigation, Methodology, Writing – original draft. TW-O: Conceptualization, Formal analysis, Methodology, Project administration, Supervision, Writing – original draft.
